# A Treatise on Poisons in Relation to Medical Jurisprudence, Physiology, and the Practice of Physic

**Published:** 1845-06

**Authors:** 


### REVIEWS.

Art. IV.—*A Treatise on Poisons in relation to Medical Jurisprudence, Physiology and the Practice of Physic.* By Robert Christison, M.D., F.R.S.E., Professor of Materia Medica in the University of Edinburgh; Fellow of the Royal College of Physicians, &c., &c.; Member of the American Philosophical Society—of the Royal Academy of Medicine of Paris, &c., &c., &c. First American, from the Fourth Edinburgh Edition. Philadelphia: Ed. Barrington & Geo. D. Haswell. 1845. 8vo. pp. 756.

[concluded.]

It is not our purpose to extend our remarks on this learned work much further, but a few points which we had not room in our last number to refer to we wish to notice briefly before taking leave of the subject. These will be found in the first part of the treatise, to which our remarks must be confined. If we should attempt to give an analysis of the second part, in which individual poisons are considered, we would be obliged to write a volume nearly as large as the one under consideration. For all that relates to the action of the several articles employed as poisons, their detection by chemical tests, and their treatment, we must refer to the treatise itself.

Our last article closed in the middle of the subject of the *moral evidence* of poisoning. We take it up here, and add some more curious illustrations of the devices to which criminals sometimes resort, and the excuses which they occasionally offer under circumstances of the sort.

Cases are recorded in which while the poisoning was confessed, a plea of ignorance was set up. The following very interesting case in which a mistake was urged by the accused, is given by our author:

"Sometimes it has been pleaded by the prisoner that he gave the poison by mistake. In all such cases, if he descends to particulars, which he cannot help doing, there is every likelihood that the falsehood of the defence will be made evident by the particulars of the story not agreeing with other particulars of the moral or medical evidence. At present it is only necessary to allude to inconsistencies in his story with the medical facts. No general rules can be laid down on the method of investigating a case with a view to evidence of this kind: I must be satisfied with an illustration from an actual occurence. On the trial of Mr. Hodgson, a surgeon, at the Durham Autumn Assizes in 1824, for attempting to poison his wife, it was clearly proved, that pills containing corrosive sublimate, and compounded by the prisoner, were given by him to her in place of pills of calomel and opium; which had been ordered by her physician. But it was pleaded by him, that being at the time intoxicated, he had mistaken, for the shop-bottle which contained opium, the corrosive sublimate bottle which stood next it. This was certainly an improbable error, considering the opium was in powder, and the sublimate in crystals. But it was not the only one which he alleged he had committed. Not long after his wife took ill, the physician sent the prisoner to the shop to prepare for her a laudnum draught, with water for the menstruum. When the prisoner returned with it, the physician, in consequence of observing it to be muddy, was led to taste it, before he gave it to the sick lady: and finding it had the taste of corrosive sublimate, he preserved it, analyzed it, and discovered that it did contain that poison. The prisoner stated in defence that he had a second time committed a mistake, and instead of water had accidentally used for the menstruum a corrosive sublimate injection, which he had previously prepared for a sailor. This was proved to have been impossible; for the injection contained only five grains to the ounce, while the draught, which did not exceed one ounce, contained fourteen grains."

The simultaneous illness of a number of persons shortly after a meal is a circumstance which will always awaken suspicion of poisoning, unless there be an epidemic prevalent, such, for example, as cholera, the symptoms of which simulate those of acrid poisoning. We remember an instance of this kind. A family consisting of eight or ten members was siezed with nausea and vomiting in a few minutes after dinner, and as cholera had been prevailing epidemically in the country, only a short time before, the sudden and alarming illness was ascribed to that disease. The physician of the family was summoned in great haste, and his fears at first were that the terrible epidemic had returned to the neighborhood; but on examination of the matters ejected from the stomach he found appearances of healthy bile, a thing not met with in the first stage of cholera, and his diagnosis, consequently, was that the illness had its origin in some other cause. This declaration set the various individuals to inquiring what that cause could be, and in the end it was discovered that the cook, in preparing the dressing for a roast turkey, had fallen unluckily upon an ounce or two of tartar emetic ointment which had been set in the cupboard in a saucer. The cook having taken of the dressing more freely than any of the rest was more violently affected, but by the use of cordials all were quickly relieved of the symptoms. In this case no chemical analysis was necessary; the moral evidence was sufficient.

The evidence of poisoning becomes very strong when it appears that all who ate of a particular dish suffered, and all who did not escaped. This point, as usual, has been strikingly illustrated by our author in the following cases:

"At other times it happens that the several people affected, suffer in proportion to the quantity taken by each of a particular dish. Too much importance ought not to be attached to the absence of that relation; for it has been already mentioned that habit, idiosyncrasy, and the state of fullness of the stomach at the time, will modify materially the action of poisons. But when present, it will often form strong evidence. A good illustration of what is now said may be found in the case of Thomas Lenargan, tried in Ireland for the murder of his master, Mr. O'Flaherty. He had for some time carried on an amour with O'Flaherty's wife; and afterwards to get rid of the troublesome surveillance of the husband, contrived to despatch him by poison. The crime was not suspected for two years. Among the facts brought out on the trial the most pointed were, that O'Flaherty's daughter and two servants were affected at the same time with the very same symptoms as himself; that they had partaken of the same dish with him; that the severity of their several complaints was in proportion to the quantity each had taken; and that others of the family, who did not eat it, were not affected.

"Another remarkable instance of this kind has been recorded by Morgagni. A clergyman, while travelling in company with another gentleman and two ladies, was setting out one afternoon to resume his journey after dining at an inn, when he was suddenly taken ill with violent pain in the stomach and bowels, and soon after with vomiting and purging. One of the ladies was similarly affected, but in a less degree; and likewise the other gentleman, though in a degree still less: but the other lady did not suffer at all. Morgagni found that this lady was the only one of the party who had not tasted a dish of soup at the commencement of dinner. But he was puzzled on finding that the gentleman who suffered least had taken the largest share of the soup, while the clergyman had taken less than either of the two that were seized along with him. He then remembered, however, that in the district where the accident happened, it was the custom to use scraped cheese with the soup in question; and on inquiry he was informed that they had each added to the soup a quantity of cheese proportioned to the severity of their illness. Here, therefore, Morgagni was led to suspect the presence of poison; and accordingly, after the whole party had fortunately recovered, the innkeeper acknowledged, that in the hurry of preparation, he had served up to his guests cheese seasoned with arsenic to poison rats."

In the case which follows we have an instance of the patient scrutiny which is often demanded if we would come at the truth in such circumstances. The author says—

"Cases of this nature are so instructive that no apology need be made for mentioning one example more which lately came under my own notice. In the case of Mary Anne Alcorn convicted here in the summer of 1827, of having administered poison to her master and mistress, it was proved that a white powder was introduced in a suspicious manner into the gravy of baked beef, which gravy was subsequently poured over the beef. Now the master of the family dined heartily on beef, potatoes and rice-pudding, and mixed the greater part of the beef gravy with his pudding; the mistress ate moderately of the first slices of the beef, took very little gravy, even to the beef, and none at all to the pudding; a little girl, their niece, dined on pudding alone, without gravy; and the prisoner dined after the family on beef and potatoes. Accordingly the master suffered so severely as for two or three days to be in danger of his life, the mistress was also severely, but by no means so violently affected, the little girl did not suffer at all, and the servant had merely slight pain and sickness at stomach. The evidence thus produced was exceedingly strong, more particularly when coupled with the fact, that the beef used was half of a piece, the other half of which had been used by the family two days before, without any ill consequences."

These quotations will give the reader a just idea of the interest of this work, but no conception, we are fully aware, of the accurate scientific information in which it abounds. That can only be appreciated by a careful perusal, or by a reference to its pages, in some case of alleged poisoning, for instruction as to the tests applicable in such cases. We shall not attempt to follow the author into the second part of his work, but in closing give the following statistics which will be regarded with interest.

"It may be well to point out in the first instance the poisons in most general use. These will appear from the following tables. The first is compiled from a Parliamentary Return of the cases of fatal poisoning brought before the coroners of England in two years ending with 1838.

| | | | |
|---|---|---|---|
| 1. *Arsenical.* | White arsenic, | 185 | |
| | Yellow arsenic, | 1 | |
| | | | 186 |
| 2. *Acids.* | Sulphuric acid, | 32 | |
| | Nitric acid, | 3 | |
| | Oxalic acid, | 19 | |
| | | | 54 |
| 3. *Mercurials.* | Corrosive sublimate, | 12 | |
| | White mercury, | 1 | |
| | Turbith-mineral, | 1 | |
| | Mercury (?) | 1 | |
| | | | 15 |
| 4. *Other mineral irritants.* | Tartar emetic, | 2 | |
| | Sulphate of iron, | 1 | |
| | Chloride of tin, | 1 | |
| | Subacetate of lead, | 1 | |
| | Bichrom. of potash, | 1 | |
| | Percussion powder, | 1 | |
| | Carbonate of potash, | 1 | |
| | Black-ash, | 1 | |
| | | | 9 |
| 5. *Veget. irritants.* | Colchicum, | 3 | |
| | Hellebore, | 1 | |
| | Savin, | 1 | |
| | Cayenne, | 1 | |
| | Castor seeds, | 1 | |
| | Morison pills, | 1 | |
| | | | 8 |
| 6. *Anim. irrits.* | Cantharides, | | 2 |
| 7. *Opium.* | Opium or Laudan. | 180 | |
| | Opium & nit. acid, | 1 | |
| | Poppy-syrup, | 4 | |
| | Godfrey's Cordial, | 6 | |
| | Morphia, | 1 | |
| | Acetate of morphia, | 1 | |
| | | | 183 |
| 8. *Hydrocyanic acid,* | Med. Hydroc. acid, | 27 | |
| | do. & Laudanum, | 1 | |
| | Ess. oil of Almonds, | 5 | |
| | Bay-leaves, | 1 | |
| | | | 34 |
| 9. *Other veget. Narcotics.* | Nux-vomica, | 3 | |
| | Strychnia, | 2 | |
| | Belladonna, | 2 | |
| | Hemlock, | 1 | |
| | Monkshood, | 2 | |
| | Spirits, | 4 | |
| | Fungi, | 4 | |
| | | | 18 |
| 10. *Narcot. gases.* | Coal gas, | | 2 |
| 11. Unascertained, | | | 22 |
| | Total, | | 543 |

"In France, in seven years, from 1825 to 1831, inclusive, there were 216 trials for poisoning, at which 273 persons were charged with the crime, and only 102 condemned. In 94 cases occurring between November, 1825, and October 1832, the substances employed were as follows:

| | | | |
|---|---|---|---|
| Arsenic | 54 | Cerusse | 1 |
| Orpiment | 1 | Mercurial ointment | 1 |
| Verdigris | 7 | Cantharides | 4 |
| Corrosive Sublimate | 5 | Nux vomica | 1 |
| Fly-powder | 3 | Opium | 1 |
| Tartar emetic | 1 | Sulphuric acid | 1 |
| Sulphate of zinc | 1 | Nitric acid | 1 |
| Acetate of lead | 1 | Unascertained | 5 |

"In the subsequent seven years there were 218 trials, and 153 prisoners condemned. Among 194 of these the following were the poisons used:

| | | | |
|---|---|---|---|
| Metallic arsenic | 5 | Nitric acid | 2 |
| Arsenious acid | 132 | Muriatic acid | 1 |
| Arsenite of copper | 1 | Hydrocianic acid | 1 |
| Compounds of copper | 13 | Ammonia | 1 |
| Corrosive sublimate | 10 | Belladonna | 1 |
| Artificial orpiment | 3 | Opium | 3 |
| Sulphate of zinc | 1 | Morphia | 1 |
| Tartar emetic | 1 | Nux-vomica | 1 |
| Cerusse | 1 | Cantharides | 10 |
| Sulphuric acid | 5 | | |

"In Denmark, in five years ending with 1835, there were 99 cases of poisoning of all sorts, 16 by arsenic, 74 by sulphuric or nitric acid, 4 by potash, 1 by an unascertained caustic substance, 2 by opium, 1 by litharge, and 1 by copper. Only 5 cases, namely, 3 by arsenic and 2 by sulphuric acid, were cases of murder, or attempt to murder."

The publishers of this treatise have had it done up in two styles of binding, corresponding to its two-fold character of medical and law. It deserves the attention of both professions, and for the sake of liberal, enlightened science we could wish that it might find its way into the libraries of every lawyer and physician in the country. Y.

